# Distributed Prescribed Performance Formation Tracking for Unknown Euler–Lagrange Systems Under Input Saturation

**DOI:** 10.3390/s25196002

**Published:** 2025-09-29

**Authors:** Athanasios K. Gkesoulis, Andreani Christopoulou, Charalampos P. Bechlioulis, George C. Karras

**Affiliations:** 1 Athena Research Center, Robotics Institute, 15125 Marousi, Greece; chmpechl@athenarc.gr (C.P.B.); karrasg@athenarc.gr (G.C.K.); 2 Department of Informatics and Telecommunications, University of Thessaly, 35100 Lamia, Greece; anchristopoulou@uth.gr; 3 Department of Electrical and Computer Engineering, University of Patras, 26504 Patras, Greece

**Keywords:** formation control, uncertain systems, nonlinear systems, robust control, distributed control, constrained control

## Abstract

In this paper, we propose a distributed prescribed performance formation tracking control method for unknown Euler–Lagrange systems subject to input amplitude constraints. We address the challenge of maintaining formation tracking within predefined performance bounds when the agents’ inputs are subject to saturation. This is achieved by designing a distributed virtual velocity reference modification mechanism, which modifies the desired velocity reference of each agent whenever saturation occurs. We establish sufficient feasibility conditions for the input constraints that ensure prescribed performance formation tracking of the desired trajectory and guarantee the boundedness of all closed-loop signals. Simulations on a team of underwater vehicles validate the method’s effectiveness.

## 1. Introduction

The distributed coordination of multi-agent systems (MASs) has attracted extensive attention due to its capacity for the collaborative execution of complex tasks, resilience to individual failures, and adaptability in dynamic environments. These qualities have rendered MASs highly valuable for diverse real–world applications, such as autonomous vehicles, robotic swarms, and sensor networks [1,2]. Among numerous MAS challenges, formation tracking control is a pivotal task that ensures that the agents collectively reach and maintain a predefined formation around a leader agent, using limited, locally available information. Although formation control has been extensively studied in recent decades [3,4,5], substantial issues remain unresolved, especially for nonlinear, uncertain agent dynamics with constraints.

One critical issue is posed by inherent system nonlinearities, prevalent in practical systems due to actuator dynamics, friction, and other phenomena. Traditional linear control strategies typically fail in the aforementioned contexts, necessitating advanced methodologies that effectively compensate for these nonlinearities to ensure robust and stable performance. Several adaptive control studies have been reported to address nonlinear dynamics and uncertainties for multi-agent systems [6,7,8,9,10,11,12]. However, a fundamental issue that remains unaddressed in the aforementioned studies is the presence of input constraints resulting from actuator saturation, safety considerations, and energy limitations. Ignoring input constraints in nonlinear systems can induce significant performance deterioration or even instability, leading to integrator wind-up phenomena [13,14]. This challenge is particularly pronounced in MASs, because if an individual agent’s input saturates, the collective system’s performance is directly influenced, potentially hindering the achievement of global objectives. This issue has attracted research attention in recent years for single [15,16,17,18,19,20] and multi-agent nonlinear systems [21,22,23,24,25,26,27,28,29,30].

Another prevalent challenge in the control of nonlinear single- and multi-agent systems is to impose prescribed performance guarantees on the transient and steady-state behavior of the system, especially in the case where the system dynamics are unknown. Prescribed performance control (PPC) has emerged as a powerful control methodology capable of addressing the challenges posed by unknown nonlinearities and uncertainties. PPC guarantees stability with predefined transient and steady-state error bounds and allows for the specification of error convergence speed, maximum allowable overshoot, and maximum steady-state error even for unknown dynamics [31]. When considered in an MAS framework, PPC methodologies aim to guarantee that agents achieve group goals with predetermined transient behavior and steady-state errors for the multi-agent system despite the presence of uncertainties and nonlinear dynamics [32]. Several studies have extended PPC frameworks to handle input saturation in nonlinear single-agent systems [33,34,35,36]. These approaches, however, frequently employ computationally demanding approximation techniques, such as neural networks or observers, complicating their practical applicability. Recent research has addressed this issue by developing simpler controllers without relying on approximation schemes [37,38,39,40,41]. Specifically, methods involving adaptive adjustments of performance bounds, reference modification schemes, or switching control have proven effective for single-agent scenarios. Nonetheless, extending these solutions to multi-agent systems remains unaddressed.

Several recent studies have addressed the cooperative control problem for Euler–Lagrange multi-agent systems under input saturation. A coordinated formation containment algorithm that embeds a dynamic auxiliary system into each agent is developed in [23], ensuring actuator bounds remain independent of neighbor count while achieving simultaneous leader formation and follower containment for Euler–Lagrange systems with known dynamics. A model-independent formation-tracking scheme is introduced in [24], where formation control with disturbance rejection is achieved, assuming the nonlinear parameters of the Euler–Lagrange model are bounded. In [25], a distributed control design for the formation containment of Euler–Lagrange systems is developed utilizing linear matrix inequalities. A study [26] considered an event-triggered mechanism for Euler–Lagrange systems with known dynamics. More recently, ref. [29] presented an event-triggered adaptive protocol for formation control input, assuming bounded nonlinearities. In [30], a design employing virtual signal generators and sliding mode control is utilized to solve the cooperative control problem. However, most of the aforementioned studies impose restrictive assumptions on system dynamics and none addresses prescribed performance, i.e., guaranteeing that both transient and steady-state formation errors remain within designer-specified bounds in the presence of input saturation. Limited efforts have tackled prescribed performance formation tracking for nonlinear MASs under input constraints. The existing literature focuses on restrictive system classes, neural network approximations, or event-triggered mechanisms [42,43,44,45]. Consequently, a research gap persists regarding generalized, computationally efficient, and robust distributed control strategies that can simultaneously handle nonlinear dynamics and input saturation, as well as guarantee prescribed performance.

Motivated by the aforementioned challenges, the current paper introduces a novel low-complexity distributed PPC strategy for unknown Euler–Lagrange MASs with input constraints under directed communication. Our contributions are summarized as follows:

- We propose a new robust, approximation-free distributed PPC strategy, where each agent leverages only local distances from its neighbors to achieve formation tracking around a leader trajectory.
- A distributed virtual velocity reference modification mechanism is developed, enabling each agent to adjust its virtual velocity reference dynamically, in response to input saturation, thus preserving internal stability and feasibility.
- Analytical lower bounds for the input saturation thresholds are derived, ensuring feasibility of the proposed control law within prescribed performance constraints.
- A 3D simulation scenario for a group of unmanned underwater vehicles is provided to demonstrate the effectiveness of the distributed formation tracking control design.

Compared to the related work on the cooperative control of nonlinear systems [6,7,8,9,10,11,12], the main contributions of the present study are the adoption of input saturation and the prescribed performance guarantees. Input saturation poses significant difficulty, especially when the considered dynamics are unknown, and it can lead to deteriorated performance and even instability. An extension of the aforementioned studies to include input saturation (even without prescribed system performance) is not straightforward, if possible at all. With respect to the related studies [21,22,23,24,25,26,27,28,29,30] on nonlinear multi-agent system control, the distinctive contribution of our study is the prescribed performance guarantees for the local formation error and the local velocity error of each agent. Also, compared to the aforementioned studies, we develop a low-complexity control design that does not employ complex approximation structures, such as neural networks and fuzzy approximators, thus rendering our approach easier to implement. Compared to the most closely related studies, which consider nonlinear multi-agent systems with input saturation and prescribed performance guarantees [42,43,44,45], the distinctive contribution of the proposed method is that it addresses a larger class of systems than all of those studies. Furthermore, there exist separate contributions compared to each of them individually. In particular, ref. [42] considers a more restrictive communication network topology than our study, being undirected and connected in contrast to directed with a spanning tree. Ref. [43] has more restrictive requirements for the leader’s trajectory, assuming that more information about the leader’s position is transmitted through the communication network, i.e., its first and second derivatives compared to only the position in our study. It also employs neural network approximators. Contrasted with [44], our study assumes relaxed communications, i.e., a directed graph with a spanning tree versus a connected undirected graph. Also, the dynamics of the systems considered in [44] are assumed to be input-to-state stable, an assumption not present in this study. Finally, ref. [45] assumes that the second derivative of the leader’s trajectory is also bounded and employs neural network approximators, contrary to our study.

The remainder of this paper is structured as follows: Section 2 reviews the necessary preliminaries. Section 3 formally presents the considered problem. Section 4 details the control design and includes its stability analysis. Section 5 illustrates the effectiveness of the proposed method through simulations. Section 6 provides concluding remarks and suggests future research directions.

## 2. Preliminaries

### Graph Theory

To model the inter-agent interactions, we consider a directed graph, which is defined as $G:=\left(V,E\right)$, where V:={1,…,N} is the set of agents (nodes) and E⊆V×V is the set of communication links (edges). We denote (i,j)∈E when agent *j* receives interaction information from agent *i*. We consider that if (i,j)∈E, then the agent *j* is aware of its relative position to agent *i*. The neighborhood of *i* is defined as $N_{i}:=\left\{j|\left(j,i\right)\in E\right\}$. The Laplacian matrix of G is defined as L:=D−A, where $A:=\left[a_{i,j}\right]\in R^{N\times N}$ is the adjacency matrix, with $a_{i,j}>0$ if $j\in N_{i}$ and $a_{i,j}=0$ otherwise, and $D:=\mathrm{diag}\left(d_{1},\ldots ,d_{N}\right)\in R^{N\times N}$ is the in-degree matrix, with $d_{i}:=\sum_{j=1}^{N}a_{i,j}$. A directed path from *i* to *j* is defined as a sequence of successive edges (i,k)(k,l)…(ℓ,j). A directed graph contains a spanning tree if there exists a root node from which there exists a directed path to every other node in V.

In this study, we consider that there exists a leader node *L*. The communication between agents and the leader is defined via the matrix $B:=\mathrm{diag}\left(b_{1},\ldots ,b_{N}\right)\in R^{N\times N}$, $b_{i}\geq 0$. If $b_{i}>0$, then the agent *i* is aware of its position’s distance to the leader weighted by $b_{i}$. We can now define the augmented graph that, in addition to G, captures the interactions of the agents with the leader, as $\bar{G}:=\left(\bar{V},\bar{E}\right)$, where $\bar{V}:=\left\{L,1,\ldots ,N\right\}$ and $\bar{E}\subseteq \bar{V}\times \bar{V}$.

We restate here a useful Lemma from [46] that will be used in the text:

**Lemma 1.**  *Consider an augmented graph $\bar{G}$ that contains a spanning tree with the root being the leader L. Then the matrix L+B is non-singular and there exists a diagonal and positive definite matrix $P\in R^{N\times N}$ and a positive constant δ such that P(L+B)+(L+B)P⪰δP.*

## 3. Problem Formulation

In this section, we formally define the control problem addressed in this study. We begin by introducing the class of systems under consideration as well as the standing assumptions. We then define the desired prescribed performance tracking and the control objectives.

### 3.1. System Dynamics and Standing Assumptions

We consider *N* agents, where each agent *i* is an uncertain Euler–Lagrange system with dynamics described by the following equations:

(1) $$\begin{array}{cc} {\dot{\eta }}_{i}= & v_{i}+g_{1,i}\left(t,\eta_{i}\right) \end{array}$$

(2) $$M_{i}\left(\eta_{i}\right){\dot{v}}_{i}+C_{i}\left(\eta_{i},v_{i}\right)v_{i}+g_{2,i}\left(t,\eta_{i},v_{i}\right)=\mathrm{sat}(\tau_{i}),$$

where $\eta_{i}={\left[\eta_{i,1}\cdots \eta_{i,m}\right]}^{T}\in R^{m}$ is the generalized position vector, $v_{i}={\left[v_{i,1}\cdots v_{i,m}\right]}^{T}\in R^{m}$ is the generalized velocity, $M_{i}\left(\eta_{i}\right)\in R^{m\times m}$ is the inertia matrix, and $C_{i}\left(\eta_{i},v_{i}\right)\in R^{m\times m}$ represents Coriolis, centripetal, and drag effects. The vectors $g_{1,i}$ and $g_{2,i}$ model unknown but bounded disturbances, unmodeled dynamics, gravitational forces, and other nonlinear effects. The control input vector is denoted as $\mathrm{sat}(\tau_{i})={\left[\mathrm{sat}\left(\tau_{i,1}\right)\cdots \mathrm{sat}\left(\tau_{i,m}\right)\right]}^{T}\in R^{m}$, where $\tau_{i}={\left[\tau_{i,1}\cdots \tau_{i,m}\right]}^{T}\in R^{m}$. The continuous saturation function sat(·):R→R is defined as

(3) $$\mathrm{sat}\left(\tau_{i,j}\right)=\left\{\begin{array}{cc} \tau_{i,j}, & \mathrm{if}\;|\tau_{i,j}|<\tau_{i,j,\max}\\ \tau_{i,j,\max}\cdot \mathrm{sgn}\left(\tau_{i,j}\right), & \mathrm{otherwise}, \end{array}\right.$$

where $\tau_{i,j,\max}>0$ is the saturation level for each input component $\tau_{i,j},\;j=1,\ldots ,m$, and sgn(·) is the signum function. We make the following assumptions:

**Assumption 1.**  *All system matrices and vectors are continuous functions with respect to time and are locally Lipschitz with respect to the rest of their arguments.*

**Assumption 2.**  *The functions $g_{j,i},\;j=1,2$ are uniformly bounded with respect to time to incorporate the effect of time-dependent bounded disturbances.*

**Assumption 3.**  *The inertia matrix $M_{i}\left(\eta_{i}\right)$ is diagonal and uniformly positive definite for all $\eta_{i}\in R^{m}$.*

**Remark 1.**  *Assumption 3 facilitates the development of an approximation-free control scheme that does not rely on any knowledge or estimation of the system dynamics. In cases where the full inertia matrix is known or can be estimated, the control framework could, in principle, be extended to account for inertial coupling.*

**Assumption 4.**  *The augmented directed communication graph $\bar{G}$ contains a spanning tree with the leader node as the root.*

**Assumption 5.**  *The leader’s trajectory $\eta_{L}\left(t\right)={\left[\eta_{L,1}\left(t\right)\cdots \eta_{L,m}\left(t\right)\right]}^{T}:R_{+}\to R^{m}$ is continuously differentiable and uniformly bounded, i.e., $|\eta_{L,j}\left(t\right)|\leq {\bar{\eta }}_{L,j},\forall j=1,\ldots m$, on $R_{+}$, with a bounded, although unknown, derivative with respect to time, i.e., $|{\dot{\eta }}_{L,j}\left(t\right)|\leq {\bar{\dot{\eta }}}_{L,j},\forall j=1,\ldots m$, on $R_{+}$.*

### 3.2. Problem Definition

The objective of this study is to design a distributed control law for each agent that guarantees that the agents’ positions will converge to a predefined formation around the leader’s position while satisfying predefined transient and steady-state performance guarantees. As in the literature of distributed prescribed performance control, the performance requirement is for the formation error of each agent to remain bounded by a prescribed envelope function of the form

$$\rho_{i,p}=\left(\rho_{i,p}^{0}-\rho_{i,p}^{\infty }\right)e^{-\lambda_{i,p}t}+\rho_{i,p}^{\infty },$$

where $\rho_{i,p}^{0}=\rho_{i,p}\left(0\right)$, $\rho_{i,p}^{\infty }=\lim_{t\to \infty }\left(\rho_{i,p}\left(t\right)\right)>0$ and $\lambda_{i,p}>0$, for all i∈{1,…,N}.

In the presence of input saturation such as (3), solving the standard prescribed performance formation control problem would be equivalent to either internal instability or letting the saturation levels become arbitrarily large, defeating the purpose of the input constraints, as stated in [38] for single-agent systems. To overcome this problem, the goal of this study is to design a distributed control mechanism that will allow each agent to modify its local virtual velocity error calculation appropriately whenever the control input becomes saturated to make it feasible under saturation. The goals of this study can be delineated as follows:

1. Design a distributed mechanism that appropriately modifies the local virtual velocity error of each agent whenever its input becomes saturated.
2. Design a distributed control protocol that ensures that the modified formation tracking error adheres to the prescribed specifications.
3. The modification and control mechanism should be continuous and of low computational effort, i.e., no approximation structures, such as neural networks or fuzzy approximators, should be used.
4. Provide conditions for the saturation level of each agent that make the prescribed performance specifications feasible.

## 4. Results

In this section, we present our distributed formation tracking strategy for a group of input-constrained Euler–Lagrange agents. First, we introduce the distributed virtual velocity error modification mechanism that enables the rest of the design. The virtual velocity reference modification mechanism activates when individual actuators saturate to appropriately modify the desired virtual velocity reference. This makes the desired virtual velocity reference feasible under saturation. Building on this, we design a continuous, approximation-free distributed control law that guarantees each agent’s formation error evolves within its prescribed performance bounds. We then state and prove the main stability theorem, establishing the boundedness and convergence properties of the closed-loop system. Finally, to illustrate and verify our theoretical developments, we provide high-fidelity simulation results for a group of BlueROV2 underwater vehicles performing formation tracking under input saturation.

### 4.1. Distributed Virtual Velocity Reference Modification Mechanism

We begin by designing a local virtual velocity reference modification scheme for each agent that remains zero when the agent’s input remains unsaturated and becomes active when the agent’s input saturates. The state of this mechanism will be utilized to alter the desired virtual velocity reference for the agent in order to make it feasible for the controller to achieve when saturation occurs.

For each agent i∈V, we design a local virtual velocity reference modification signal $\sigma_{v,i}:={\left[\sigma_{v,i,1}\cdots \sigma_{v,i,m}\right]}^{T}\in R^{m}$ that is given as the state of the dynamics

(4) $$\begin{array}{cc} {\dot{\sigma }}_{v,i}\left(t\right) & =-\beta_{i}\sigma_{v,i}\left(t\right)+\Delta \tau_{i}, \end{array}$$

where $\Delta \tau_{i}:=\tau_{i}-\mathrm{sat}\left(\tau_{i}\right)$, $\sigma_{v,i}\left(0\right)=0$ and $\beta_{i}>0$ are design constants for all i∈V.

### 4.2. Control Design

The distributed control law is built upon the velocity error modification signal $\sigma_{v,i}$ introduced in (4). Specifically, each agent’s desired virtual velocity reference is augmented by $\sigma_{v,i}$ whenever its actuator saturates, thereby preserving prescribed performance in the presence of input limits. We begin by defining the local formation error for each agent, which captures both neighbor-to-neighbor and leader-to-agent deviations.

For agent i∈V and generalized coordinate j=1,…,m, let

(5) $$\begin{array}{c} {\hat{\eta }}_{i,j}:=\eta_{i,j}-c_{i,j}, \end{array}$$

where $c_{i,j}$ is the desired offset of agent *i* along coordinate *j* from the leader’s position in the formation. Then the local formation error for agent *i* in the *j*-th coordinate $e_{i,j}$ is defined by

(6) $$\begin{array}{cc} e_{i,j} & :=\sum_{\ell \in N_{i}}a_{i\ell }\left({\hat{\eta }}_{i,j}-{\hat{\eta }}_{\ell ,j}\right)+b_{i}\left({\hat{\eta }}_{i,j}-\eta_{L,j}\right), \end{array}$$

for all j=1,…,m and i=1,…,N. Here, $a_{i\ell }$ are the adjacency weights and $b_{i}\geq 0$ the leader connection weights introduced in Section 2.

The distributed control design consists of two steps:

*Step 1:* Define the normalized formation error and virtual velocity:

We first normalize the formation errors (6) by the prescribed performance functions. For each agent i=1,…,N and coordinate j=1,…,m, define the normalized formation error

(7) $$\xi_{p,i,j}\left(t\right)\;:=\frac{e_{i,j}\left(t\right)}{\rho_{p,i,j}\left(t\right)},$$

where the prescribed performance function $\rho_{p,i,j}\left(t\right)$ is any continuously differentiable, strictly decreasing function satisfying

(8) $$\begin{array}{c} \rho_{p,i,j}\left(0\right)>\left|e_{i,j}\left(0\right)\right|,\;\lim_{t\to \infty }\rho_{p,i,j}\left(t\right)=\rho_{p,i,j}^{\infty }>0. \end{array}$$

A typical choice is

(9) $$\begin{array}{cc} \rho_{p,i,j}\left(t\right)\; & =\left(\rho_{p,i,j}\left(0\right)-\rho_{p,i,j}^{\infty }\right)e^{-\lambda_{p,i,j}t}\;+\;\rho_{p,i,j}^{\infty }, \end{array}$$

with $\lambda_{p,i,j}>0$. Next, we assign each agent a virtual velocity reference that will drive $\xi_{p,i,j}$ towards zero. Let

(10) $$v_{d,i,j}\left(t\right)\;:=-\;\frac{k_{p,i,j}}{\rho_{p,i,j}\left(t\right)}\;\frac{2}{1-\xi_{p,i,j}^{2}\left(t\right)}\;T\left(\xi_{p,i,j}\left(t\right)\right),$$

where $k_{p,i,j}>0$ is a design gain and

(11) $$\begin{array}{c} T\left(s\right)\;=\;\ln\;\left(\frac{1+s}{1-s}\right),\;s\in \left(-1,1\right). \end{array}$$

*Step 2:* Define the normalized velocity error and the control law:

To incorporate actuator saturation effects, we use the modification signal $\sigma_{v,i,j}$ from (4). For each agent i=1,…,N and coordinate j=1,…,m, define the normalized velocity error

(12) $$\begin{array}{cc} \xi_{v,i,j}\left(t\right)\; & :=\frac{v_{i,j}\left(t\right)-v_{d,i,j}\left(t\right)+\sigma_{v,i,j}\left(t\right)}{\rho_{v,i,j}\left(t\right)}, \end{array}$$

where $\rho_{v,i,j}\left(t\right)$ is another prescribed performance function for the velocity error that can be chosen analogously to (9) with $\rho_{v,i,j}\left(0\right)>\left|v_{i,j}\left(0\right)-v_{d,i,j}\left(0\right)\right|$, and $\lim_{t\to \infty }\rho_{v,i,j}\left(t\right)=\rho_{v,i,j}^{\infty }>0$. Finally, for each agent i=1,…,N and coordinate j=1,…,m, select the control law components as

(13) $$\begin{array}{cc} \tau_{i,j}\left(t\right)\; & :=-\;k_{v,i,j}\;T\left(\xi_{v,i,j}\left(t\right)\right), \end{array}$$

where $k_{v,i,j}>0$ is a control gain.

**Figure 1:**
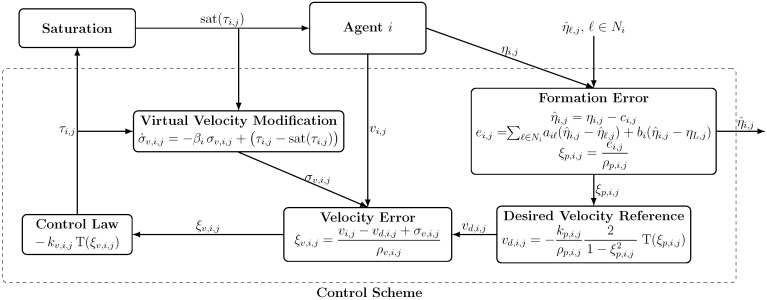
Closed-loop block diagram of the distributed prescribed performance control scheme for agent *i*.

The closed-loop signal flow of the proposed distributed control scheme is summarized in Figure 1, illustrating the signal flow and the interconnection of each component.

### 4.3. Stability Analysis

We are now ready to state our main stability result.

**Theorem 1.**  *Consider a group of N agents with dynamics given by *(1) *and* (2) *under Assumptions 1–5. The distributed control law* (13) *together with the modification mechanism* (4) *ensures that if*

(14) $$\begin{array}{c} \tau_{i,j,\max}\geq \max\left\{|\tau_{i,j}\left(0\right)|-\Lambda ,\underset{t\geq 0}{\sup}\max_{(\eta_{i},v_{i})\in S}\left[m_{i,j}\left(\eta_{i}\right)\left(|f_{i,j}\left(t,\eta_{i},v_{i}\right)|+{\tilde{\dot{v}}}_{d,i,j}+{\dot{\rho }}_{v,i,j}\right)\right]\right\}, \end{array}$$

*where Λ≥0, $f_{i}\left(t,\eta_{i},v_{i}\right):=-M_{i}^{-1}C_{i}\left(\eta_{i},v_{i}\right)\;v_{i}-M_{i}^{-1}g_{2,i}\left(t,\eta_{i},v_{i}\right)$ and S is defined in the proof, then all closed-loop signals remain bounded and prescribed performance formation is achieved, in the sense that $|e_{i,j}\left(t\right)|<\rho_{p,i,j}\left(t\right)$ for all t≥0, i=1,…,N and j=1,…,m, and*

(15) $$|{\hat{\eta }}_{i,j}-\eta_{L,j}|\leq \frac{∥R_{p,j}\left(t\right)∥}{\sigma_{\min}\left(L+B\right)}\leq \frac{\left(N^{2}+N+1\right)}{{\left(\frac{N-1}{N}\right)}^{\frac{N-1}{2}}}∥R_{p,j}\left(t\right)∥,$$

*where $1={\left[1\ldots 1\right]}^{T}$ and $R_{p,j}\left(t\right)=\mathrm{diag}\left(\rho_{p,1,j}\left(t\right),\ldots ,\rho_{p,N,j}\left(t\right)\right)$ for all j=1,…,m.*

**Proof.**  First, since each $M_{i}$ is diagonal and uniformly positive definite, we can rewrite the dynamics (2) as

(16) $${\dot{\eta }}_{i}=v_{i}+g_{1,i}\left(t,\eta_{i}\right),$$

(17) $${\dot{v}}_{i}=-M_{i}^{-1}\left(\eta_{i}\right)C_{i}\left(\eta_{i},v_{i}\right)v_{i}-M_{i}^{-1}\left(\eta_{i}\right)g_{2,i}\left(t,\eta_{i},v_{i}\right)+M_{i}^{-1}\left(\eta_{i}\right)\mathrm{sat}\left(\tau_{i}\right).$$

Now, we can rewrite (17) as

(18) $${\dot{v}}_{i}=f_{i}\left(t,\eta_{i},v_{i}\right)+M_{i}^{-1}\left(\eta_{i}\right)\mathrm{sat}\left(\tau_{i}\right).$$

Next, let us recall the definitions of the normalized errors and virtual reference

(19) $$\begin{array}{c} \xi_{p,i,j}=\frac{e_{i,j}}{\rho_{p,i,j}},\;v_{d,i,j}=-\frac{k_{p,i,j}}{\rho_{p,i,j}}\;\frac{2}{1-\xi_{p,i,j}^{2}}\;T\left(\xi_{p,i,j}\right), \end{array}$$

and set

(20) $$\begin{array}{c} {\tilde{v}}_{i,j}:=v_{i,j}-v_{d,i,j}+\sigma_{v,i,j}. \end{array}$$

Then, from (12),

(21) $$\begin{array}{c} \xi_{v,i,j}=\frac{{\tilde{v}}_{i,j}}{\rho_{v,i,j}}. \end{array}$$

Let us now differentiate $\xi_{p,i,j}$ with respect to time. First, from the definition of the formation error $e_{i,j}$, one obtains

(22) $$\begin{array}{c} {\dot{e}}_{i,j}=\sum_{\ell \in N_{i}}a_{i\ell }\left[v_{i,j}+g_{1,i,j}\left(t,\eta_{i}\right)-v_{\ell ,j}-g_{1,\ell ,j}\left(t,\eta_{\ell }\right)\right]+b_{i}\left[v_{i,j}+g_{1,i,j}\left(t,\eta_{i}\right)-{\dot{\eta }}_{L,j}\right]. \end{array}$$

Hence, using ${\dot{\xi }}_{p,i,j}=\left({\dot{e}}_{i,j}-{\dot{\rho }}_{p,i,j}\;\xi_{p,i,j}\right)/\rho_{p,i,j}$, we have

(23) $$\begin{array}{cc} {\dot{\xi }}_{p,i,j}\left(t\right)=\frac{1}{\rho_{p,i,j}} & \left\{\sum_{\ell \in N_{i}}a_{i\ell }\left[v_{i,j}+g_{1,i,j}\left(t,\eta_{i}\right)-v_{\ell ,j}-g_{1,\ell ,j}\left(t,\eta_{\ell }\right)\right]\;+b_{i}\left[v_{i,j}+g_{1,i,j}\left(t,\eta_{i}\right)-{\dot{\eta }}_{L,j}\right]-{\dot{\rho }}_{v,i,j}\;\xi_{v,i,j}\right\} \end{array}$$

Differentiating $\xi_{v,i,j}$ and substituting (18) and the derivative $\sigma_{v,i,j}$ from (4) yields

(24) $$\begin{array}{cc} {\dot{\xi }}_{v,i,j} & =\frac{1}{\rho_{v,i,j}}\left\{f_{i,j}\left(t,\eta_{i},v_{i}\right)+\frac{1}{m_{i,j}\left(\eta_{i}\right)}\mathrm{sat}\left(\tau_{i,j}\right)-{\dot{v}}_{d,i,j}-\beta_{i}\;\sigma_{v,i,j}+\Delta \tau_{i,j}-{\dot{\rho }}_{v,i,j}\;\xi_{v,i,j}\right\}. \end{array}$$

Consider now the augmented state vector

(25) $$\begin{array}{c} \xi :={\left[\begin{array}{ccccccccc} \xi_{p,1}^{T} & \ldots & \xi_{p,N}^{T} & \xi_{v,1}^{T} & \ldots & \xi_{v,N}^{T} & \sigma_{v,1}^{T} & \ldots & \sigma_{v,N}^{T} \end{array}\right]}^{T}\in R^{3mN}, \end{array}$$

where for each i=1,…,N,

(26) $$\xi_{p,i}:={\left[\begin{array}{ccc} \xi_{p,i,1} & \ldots & \xi_{p,i,m} \end{array}\right]}^{T},\;\xi_{v,i}:={\left[\begin{array}{ccc} \xi_{v,i,1} & \ldots & \xi_{v,i,m} \end{array}\right]}^{T}.$$

From (4), (23), and (23), the overall closed-loop dynamics can be written as

(27) $$\dot{\xi }\;=\;H\left(t,\xi \right).$$

Let

(28) $$\begin{array}{c} \Omega_{\xi }:={\left(-1,1\right)}^{2mN}\times {\left(-{\tilde{\sigma }}_{v,i,j},{\tilde{\sigma }}_{v,i,j}\right)}^{mN} \end{array}$$

for some constants ${\tilde{\sigma }}_{v,i,j}>0$ to be defined later in the proof. $\Omega_{\xi }$ is nonempty and open. By construction of the performance functions and the reference velocity error modification mechanism, the initial condition of the augmented state satisfies

(29) $$\begin{array}{c} \xi \left(0\right)\in \Omega_{\xi }. \end{array}$$

Moreover, H(t,ξ) is continuous in *t* and locally Lipschitz in ξ on $\Omega_{\xi }$. Therefore, according to the existence and uniqueness theorem (see Th. 3.1., pp. 88, ref. [47]), there exists a unique maximal solution

(30) $$\begin{array}{c} \xi :\left[0,\;t_{\max}\right)\;⟶\;\Omega_{\xi },\;\mathrm{with}\;\;\xi \left(t\right)\in \Omega_{\xi },\;\forall \;t\in \left[0,t_{\max}\right). \end{array}$$

This implies the existence of a constant Λ≥0 such that

(31) $$\begin{array}{c} |\tau_{i,j}\left(0\right)|\leq \Lambda +\tau_{i,j,\max}, \end{array}$$

for all $t\in [0,t_{\max})$, i=1,…,N and j=1,…,m. Hence,

(32) $$\begin{array}{c} \left|T(\xi_{v,i,j}\left(0\right))\right|\leq \frac{\Lambda +\tau_{i,j,\max}}{k_{v,i,j}} \end{array}$$

and

(33) $$\begin{array}{c} |\xi_{v,i,j}\left(0\right)|\leq T^{-1}\;\left(\frac{\Lambda +\tau_{i,j,\max}}{k_{v,i,j}}\right)=:{\tilde{\xi }}_{v,i,j}, \end{array}$$

for all $t\in [0,t_{\max})$, i=1,…,N and j=1,…,m. Consequently, for $t\in [0,t_{\max})$, it holds true that

(34) $$\begin{array}{c} |\Delta \tau_{i,j}\left(t\right)|\leq \Lambda , \end{array}$$

which implies from (4) that

(35) $$\begin{array}{c} \left|\sigma_{v,i,j}\left(t\right)\right|\leq \frac{\Lambda }{\beta_{i}}=:{\tilde{\sigma }}_{v,i,j}. \end{array}$$

Let us now define the column vector

(36) $$\begin{array}{c} \epsilon_{p,j}:=\left[\begin{array}{c} T(\xi_{p,1,j})\\ T(\xi_{p,2,j})\\ \vdots \\ T(\xi_{p,N,j}) \end{array}\right], \end{array}$$

and consider the function

(37) $$\begin{array}{c} V_{p,j}:=\frac{1}{2}\;\epsilon_{p,j}^{T}P\;\epsilon_{p,j}, \end{array}$$

where *P* is the diagonal positive-definite matrix from Lemma 1. Its time derivative is

(38) $$\begin{array}{c} {\dot{V}}_{p,j}=\epsilon_{p,j}^{T}\;P\;J\left(\xi_{p,j}\right)\left[\begin{array}{c} {\dot{\xi }}_{p,1,j}\\ {\dot{\xi }}_{p,2,j}\\ \vdots \\ {\dot{\xi }}_{p,N,j} \end{array}\right], \end{array}$$

where

(39) $$\begin{array}{c} J\left(\xi_{p,j}\right):=\mathrm{diag}\left(J\left(\xi_{p,1,j}\right),\ldots ,J\left(\xi_{p,N,j}\right)\right),\;J\left(\xi_{p,i,j}\right):=\frac{2}{1-\xi_{p,i,j}^{2}}. \end{array}$$

Substituting ${\dot{\xi }}_{p,i,j}$ from (23) and

(40) $$\begin{array}{c} v_{i,j}=-\frac{k_{p,i,j}}{\rho_{p,i,j}}\;J\left(\xi_{p,i,j}\right)\;T\left(\xi_{p,i,j}\right)\;-\;\sigma_{v,i,j}\;+\;\xi_{v,i,j}\;\rho_{v,i,j}, \end{array}$$

and after some algebraic manipulations, we obtain

(41) $$\begin{array}{cc} {\dot{V}}_{p,j}\; & =\;\epsilon_{p,j}^{T}J\left(\xi_{p,j}\right)\;R_{p,j}^{-1}\left(t\right)\;P\;[\left(L+B\right){\mathrm{col}}_{i=1}^{N}\;\left(\xi_{v,i,j}\;\rho_{v,i,j}-\sigma_{v,i,j}-{\dot{\eta }}_{L,j}+g_{1,i,j}\left(t,\eta_{i}\right)\right)\\ \; & \;\;\;-K_{p,j}\;\left(L+B\right)\;R_{p,j}^{-1}\left(t\right)\;J\left(\xi_{p,j}\right)\;\epsilon_{p,j}-{\mathrm{col}}_{i=1}^{N}\;\left({\dot{\rho }}_{p,i,j}\;\xi_{p,i,j}\right)], \end{array}$$

where ${\mathrm{col}}_{i=1}^{N}\left(\cdot \right)$ is the column vector containing the indicated scalars, $K_{p,j}:=\mathrm{diag}(k_{p,1,j},\ldots ,$$k_{p,N,j})$ and $R_{p,j}\left(t\right):=\mathrm{diag}\left(\rho_{p,1,j},\ldots ,\rho_{p,N,j}\right)$. Let us define

(42) $$\begin{array}{c} w_{p,j}:=\left(L+B\right){\mathrm{col}}_{i=1}^{N}\;\left(\xi_{v,i,j}\;\rho_{v,i,j}-\sigma_{v,i,j}-{\dot{\eta }}_{L,j}+g_{1,i,j}\left(t,\eta_{i}\right)\right)\;-\;{\mathrm{col}}_{i=1}^{N}\left({\dot{\rho }}_{p,i,j}\;\xi_{p,i,j}\right). \end{array}$$

Substituting this in (41), we can obtain

(43) $$\begin{array}{cc} {\dot{V}}_{p,j}\; & \leq \;-\frac{1}{2}\;\epsilon_{p,j}^{T}J\left(\xi_{p,j}\right)\;R_{p,j}^{-1}\left(t\right)\left[P\;K_{p,j}\left(L+B\right)+{\left(L+B\right)}^{T}P\;K_{p,j}\right]R_{p,j}^{-1}\left(t\right)\;J\left(\xi_{p,j}\right)\;\epsilon_{p,j}\\ \; & \;+\;\left|\epsilon_{p,j}^{T}J\left(\xi_{p,j}\right)\;R_{p,j}^{-1}\left(t\right)P\;w_{p,j}\right|. \end{array}$$

Completing the squares and invoking Lemma 1, we have

(44) $$\begin{array}{cc} {\dot{V}}_{p,j}\; & \leq \;-\frac{\delta }{4}\;\epsilon_{p,j}^{T}J\left(\xi_{p,j}\right)\;R_{p,j}^{-1}\left(t\right)P\;K_{p,j}\;R_{p,j}^{-1}\left(t\right)J\left(\xi_{p,j}\right)\;\epsilon_{p,j}\;+\;\frac{1}{\delta }\;w_{p,j}^{T}K_{p,j}^{-1}P\;w_{p,j}. \end{array}$$

Since *P* and $K_{p,j}$ are diagonal positive-definite, we can write $PK_{p,j}=\sqrt{K_{p,j}}P\sqrt{K_{p,j}}$, where $\sqrt{K_{p,j}}=\mathrm{diag}\left(\sqrt{k_{p,1,j}},\ldots ,\sqrt{k_{p,N,j}}\right)$ and therefore

(45) $$\begin{array}{cc} \epsilon_{p,j}^{T}J\left(\xi_{p,j}\right)\;R_{p,j}^{-1}\left(t\right)P\;K_{p,j}R_{p,j}^{-1}\left(t\right) & J\left(\xi_{p,j}\right)\;\epsilon_{p,j}\;\geq \;\\ \; & \lambda_{\min}\;\left(R_{p,j}^{-1}\left(t\right)J^{-1}\left(\xi_{p,j}\right)K_{p,j}J^{-1}\left(\xi_{p,j}\right)R_{p,j}^{-1}\left(t\right)\right)\;\epsilon_{p,j}^{T}P\;\epsilon_{p,j}, \end{array}$$

where $\lambda_{\min}$ denotes the minimum eigenvalue. It follows that

(46) $$\begin{array}{c} {\dot{V}}_{p,j}\leq -\frac{\delta }{4\;\lambda_{\min}\left(R_{p,j}^{-1}\left(t\right)J^{-1}\left(\xi_{p,j}\right)K_{p,j}J^{-1}\left(\xi_{p,j}\right)R_{p,j}^{-1}\left(t\right)\right)}\;V_{p,j}\;+\;\frac{1}{\delta }\;w_{p,j}^{T}P\;w_{p,j}. \end{array}$$

Therefore, we have that ${\dot{V}}_{p,j}\leq 0$ whenever

(47) $$V_{p,j}\geq \frac{4\lambda_{\min}\left(R_{p,j}^{-1}\left(t\right)J^{-1}\left(\xi_{p,j}\right)K_{p,j}J^{-1}\left(\xi_{p,j}\right)R_{p,j}^{-1}\left(t\right)\right)}{\delta^{2}}w_{p,j}^{T}PK_{p,j}J^{-1}\;w_{p,j}.$$

Therefore, there exist constants ${\bar{\epsilon }}_{p,j}>0$ such that $∥\epsilon_{p,j}∥\leq {\bar{\epsilon }}_{p,j}$ for all $t\in [0,t_{\max})$. From the definition of $\epsilon_{p,j}$ in (36), this implies that $T(\xi_{p,i,j})$ remains bounded and, therefore, from (19), the normalized position errors remain bounded as $\left|\xi_{p,i,j}\left(t\right)\right|\leq 1$ and the virtual velocity reference and its time derivative remain bounded by some constants ${\tilde{v}}_{i,j}>0$ and ${\tilde{v}}_{d,i,j}>0$, respectively, for all $t\in [0,t_{\max})$. Since the preceding analysis is independent of *i* and *j*, the corresponding results are valid for all i=1,…,N and j=1,…,m. Equations (6) and (7), the definition of the prescribed performance functions $\rho_{p,i,j}$ and Assumptions 4 and 5, in turn, imply that there exist positive constants ${\tilde{\eta }}_{i,j}$ such that $|\eta_{i,j}|<{\tilde{\eta }}_{i,j}$ for all $t\in [0,t_{\max})$, i=1,…,N and j=1,…,m.

Next, we distinguish two cases as follows: *Case* 1*:* If $|\tau_{i,j}|<\tau_{i,j,\max}$, then $\left|T(\xi_{v,i,j}\left(t\right))\right|<\frac{\tau_{i,j,\max}}{k_{v,i,j}}\leq T\left(\xi_{v,i,j}\left(0\right)\right)$ and therefore $|\xi_{v,i,j}\left(t\right)|\leq {\tilde{\xi }}_{v,i,j}$. *Case* 2*:* If $|\tau_{i,j}|\geq \tau_{i,j,\max}$, let us consider the function

(48) $$V_{v,i,j}=\frac{1}{2}T{\left(\xi_{v,i,j}\right)}^{2}$$

Its time derivative is

(49) $$\begin{array}{cc} {\dot{V}}_{v,i,j}=\frac{2T(\xi_{v,i,j})}{\left(1-\xi_{v,i,j}^{2}\right)\rho_{v,i,j}\left(t\right)} & [f_{i,j}\left(t,\eta_{i},v_{i}\right)+\frac{1}{m_{i,j}\left(\eta_{i}\right)}\mathrm{sat}\left(\tau_{i,j}\right)\\ \; & -{\dot{v}}_{d,i,j}-\beta_{i}\;\sigma_{v,i,j}+\Delta \tau_{i,j}-{\dot{\rho }}_{v,i,j}\;\xi_{v,i,j}]. \end{array}$$

It is valid that $\mathrm{sat}\left(\tau_{i,j}\right)=-\mathrm{sgn}\left(T(\xi_{v,i,j})\right)\tau_{i,j,\max}$ and also that in the limit case that $T(\xi_{v,i,j})$ reaches its initial bound, i.e., $|T\left(\xi_{v,i,j}\right)|=\frac{\Lambda +\tau_{i,j,\max}}{k_{v,i,j}}$; it stands that $\Delta \tau_{i,j}=-\mathrm{sgn}\left(T(\xi_{v,i,j})\right)\Lambda$. Therefore, utilizing these, invoking (35) and $|\xi_{v,i,j,}\left(t\right)|<1$ from the existence and uniqueness theorem for all $t\in [0,t_{\max})$, we can obtain

(50) $$\begin{array}{c} {\dot{V}}_{v,i,j}\leq \frac{2|T(\xi_{v,i,j})|}{\left(1-\xi_{v,i,j}^{2}\right)\rho_{v,i,j}\left(t\right)}\left[|f_{i,j}\left(t,\eta_{i},v_{i}\right)|-\frac{1}{m_{i,j}\left(\eta_{i}\right)}\tau_{i,j,\max}+{\tilde{\dot{v}}}_{d,i,j}+{\dot{\rho }}_{v,i,j}\right], \end{array}$$

where ${\tilde{\dot{v}}}_{d,i,j}>0$ denotes the bound of the derivative of the virtual velocity reference. As a result, if

(51) $$\begin{array}{c} \tau_{i,j,\max}\geq \max\left\{|\tau_{i,j}\left(0\right)|-\Lambda ,\underset{t\geq 0}{\sup}\max_{(\eta_{i},v_{i})\in S}\left[m_{i,j}\left(\eta_{i}\right)\left(|f_{i,j}\left(t,\eta_{i},v_{i}\right)|+{\tilde{\dot{v}}}_{d,i,j}+{\dot{\rho }}_{v,i,j}\right)\right]\right\}, \end{array}$$

where $S=\{\left(\eta_{i},v_{i}\right)\in R^{2m}|\eta_{i,j}|\leq {\tilde{\eta }}_{i,j},|v_{i,j}|\leq \rho_{v,i,j}\left(0\right)+\frac{\Lambda }{\beta_{i}}+{\tilde{v}}_{d,i,j},j=1,\ldots ,m\}$, then ${\dot{V}}_{v,i,j}\leq 0$ and consequently $T(\xi_{v,i,j}\left(t\right))$ will remain within their initial bounds $T\left(\xi_{v,i,j}\left(t\right)\right)<\frac{\Lambda +\tau_{i,j,\max}}{k_{v,i,j}}$. Thus, we also have $|\xi_{v,i,j}\left(t\right)|\leq T^{-1}\left(\frac{\Lambda +\tau_{i,j,\max}}{k_{v,i,j}}\right)={\tilde{\xi }}_{v,i,j}<1$ for all $t\in [0,t_{\max})$. By virtue of (33), it holds that $\xi_{v,i,j}\left(0\right)\in \left[-{\tilde{\xi }}_{v,i,j},{\tilde{\xi }}_{v,i,j}\right]\subset \left(-1,1\right)$. Since $\tau_{i,j}$ is bounded, we can deduce that $|\Delta \tau_{i,j}\left(t\right)|=|\tau_{i,j}\left(t\right)-\mathrm{sat}\left(\tau_{i,j}\left(t\right)\right)|\leq \sup_{t\geq 0}\left|\Delta \tau_{i,j}\left(t\right)\right|$ and as a result, $|\sigma_{v,i,j}\left(t\right)|\leq \frac{\sup_{t\geq 0}\left|\Delta \tau_{i,j}\left(t\right)\right|}{\beta_{i}}\left(1-e^{-\beta_{i}t}\right)$ for all $t\in [0,t_{\max})$. Invoking Proposition 1 in [48], if $t_{\max}<\infty$, then there would exist $t^{\prime }\in \left[0,t_{\max}\right)$ such that $\xi_{v,i,j}\left(t^{\prime }\right)\notin \left[-{\tilde{\xi }}_{v,i,j},{\tilde{\xi }}_{v,i,j}\right]$, which is a contradiction. As a result, $t_{\max}=\infty$. The preceding analysis is independent of *i* and *j* and therefore the corresponding results are valid for all i=1,…,N and j=1,…,m. This implies that all closed-loop signals remain bounded for all t≥0.

From (7), we obtain $|e_{i,j}|\;<\rho_{p,i,j}\left(t\right)$ for all t≥0, i=1,…,N and j=1,…,m. Invoking that (L+B) is invertible from Assumption A4, we conclude that prescribed performance formation is achieved in the sense that

(52) $$∥\left(L+B\right)∥∥{\hat{\eta }}_{j}-\eta_{L,j}1∥\;\leq \;∥R_{p,j}\left(t\right)∥,$$

where ${\hat{\eta }}_{j}={\left[{\hat{\eta }}_{1,j}\ldots {\hat{\eta }}_{N,j}\right]}^{T}$ and therefore invoking Remark 2 in [32], we obtain

(53) $$|{\hat{\eta }}_{i,j}-\eta_{L,j}|\;\leq \;∥{\hat{\eta }}_{j}-\eta_{L,j}1∥\;\leq \;\frac{∥R_{p,j}\left(t\right)∥}{\sigma_{\min}\left(L+B\right)}\leq \frac{\left(N^{2}+N+1\right)}{{\left(\frac{N-1}{N}\right)}^{\frac{N-1}{2}}}∥R_{p,j}\left(t\right)∥,$$

for all t≥0, i=1,…,N and j=1,…,m, where $\sigma_{\min}\left(L+B\right)$ denotes the smallest singular value of (L+B). □

**Remark 2.**  *The parameter* Λ *in Theorem 1 is an upper bound on the difference between the requested control effort and the actual control effort that might arise in the closed-loop system.*

**Remark 3.**  *The proposed adaptive PPC algorithm operates without any knowledge of the plant nonlinearities and without using any universal approximator such as neural networks or fuzzy systems. Both the control input and the virtual velocity error modification are obtained in a straightforward manner, keeping computational overhead low. In addition, the method does not require the time derivative of the leader’s signal $\eta_{L}\left(t\right)$, which makes it suitable for applications where the desired trajectory is measured online and its closed form is not available.*

## 5. Simulation

This section provides simulation results to verify the effectiveness of the proposed control scheme.

### 5.1. Simulation Scenario

To demonstrate the effectiveness and robustness of the proposed distributed control framework, we present simulation results for a group of five BlueROV2 autonomous underwater vehicles (AUVs) operating in a six-DOF leader–follower formation, where the leader follows a sinusoidal trajectory. Each agent is modeled by full rigid-body dynamics with hydrodynamic effects using state vectors

(54) $$\begin{array}{c} \eta ={\left[x,y,z,\phi ,\theta ,\psi \right]}^{T},\;v={\left[u,v,w,p,q,r\right]}^{T} \end{array}$$

and control inputs

(55) $$\begin{array}{c} \tau ={\left[X,Y,Z,K,M,N\right]}^{T}. \end{array}$$

The inertia matrix is defined as

(56) $$\begin{array}{c} M=\mathrm{diag}(17,24.2,26.07,0.28,0.28,0.28), \end{array}$$

and the Coriolis, centripetal, and hydrodynamic drag forces matrix is modeled as

$$\begin{array}{cc} C= & \left[\begin{array}{ccc} -4.03-18.18|u| & 0 & 0\\ 0 & -6.22-21.66|v| & 0\\ 0 & 0 & -5.18-36.99|w|\\ 0 & -3.07w & 1.2v\\ 3.07w & 0 & 6u\\ -1.2v & 6u & 0 \end{array}\right.\\ \; & \;\left.\begin{array}{ccc} 0 & -3.07w & 1.2v\\ 3.07w & 0 & 6u\\ -1.2v & -6u & 0\\ -0.07-1.55|p| & -0.28r & -0.04q\\ 0.28r & -0.07-1.55|q| & 0.04p\\ 0.04q & -0.04p & -0.07-1.55|r| \end{array}\right]. \end{array}$$

The gravitational and buoyancy effects are encoded using the restoring force vector

(57) $$\begin{array}{c} g_{2}={\left[2\sin\left(\theta \right),-2\cos\left(\theta \right)\sin\left(\phi \right),-2\cos\left(\theta \right)\cos\left(\phi \right),0,0,0\right]}^{T}. \end{array}$$

The desired position offsets of the five AUVs relative to the leader’s trajectory along each coordinate *x*, *y*, *z*, ψ, and ϕ are given by the following matrix

F=−3−3000−2−2000−1−1000−−11000−−2.52.5000,

where $c_{i,j}:={\left\{F\right\}}_{ij}$, as defined in Equation (5), for *j* equal to *x*, *y*, *z*, ψ and ϕ. We note that since the BlueROV2 AUVs are not actuated in the pitch dimension (θ), we do not present a control design and results for this dimension.

The prescribed performance functions were selected for all agents i=1,…,5 as

- $\rho_{p,i,j}\left(t\right)=\left(\rho_{p,i,j}^{0}-\rho_{p,i,j}^{\infty }\right)e^{-\lambda_{p,i,j}t}+\rho_{p,i,j}^{\infty },\;\forall j=x,y,z,\phi ,\psi$;
- $\rho_{v,i,j}\left(t\right)=\left(\rho_{v,i,j}^{0}-\rho_{v,i,j}^{\infty }\right)e^{-\lambda_{v,i,j}t}+\rho_{v,i,j}^{\infty },\;\forall j=u,v,w,p,r$;

with performance parameters

- $\rho_{p,i,j}^{0}=30,\;\rho_{p,i,j}^{\infty }=0.001,\;\lambda_{p,i,j}=0.01\;\forall j=x,y,z,\phi ,\psi$;
- $\rho_{v,i,j}^{0}=30,\;\rho_{v,i,j}^{\infty }=0.5,\;\lambda_{v,i,j}=0.05\;\forall j=u,v,w,p,r$.

Control gains were chosen as

- $k_{p,i,j}=50,\;\forall j=x,y,z,\phi ,\psi$;
- $k_{v,i,j}=1,\;\forall j=u,v,w,p,r$.

The virtual velocity reference modification gains were selected as $\beta_{i}=0.5$ for all i=1,…,5.

The control inputs were constrained by saturation limits

(58) $$\begin{array}{c} \tau_{x,\max}=5,\;\tau_{y,\max}=5,\;\tau_{z,\max}=5,\;\tau_{\phi ,\max}=0.5,\;\tau_{\psi ,\max}=0.5. \end{array}$$

The desired leader trajectory was designed as

(59) $$\begin{array}{cc} \eta_{L,x}\left(t\right) & =10\sin\left(0.01t\right), \end{array}$$

(60) $$\begin{array}{cc} \eta_{L,y}\left(t\right) & =10\sin\left(0.02t\right), \end{array}$$

(61) $$\begin{array}{cc} \eta_{L,z}\left(t\right) & =5\sin\left(0.03t\right), \end{array}$$

(62) $$\begin{array}{cc} \eta_{L,\phi }\left(t\right) & =\frac{\pi }{6}\sin\left(0.004t\right), \end{array}$$

(63) $$\begin{array}{cc} \eta_{L,\psi }\left(t\right) & =\frac{\pi }{2}\sin\left(0.004t\right). \end{array}$$

**Figure 2:**
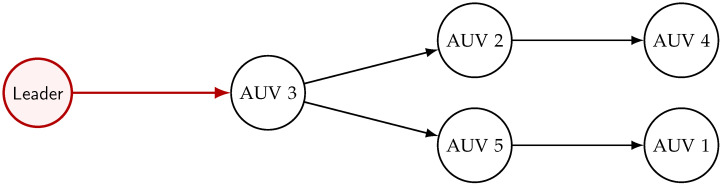
The communication graph topology.

The multi-AUV system is configured in a topology with a spanning tree with the leader as the root. The communication topology of the group of AUVs is depicted in Figure 2. We can observe that only AUV 3 is aware of its relative position to the leader.

### 5.2. Simulation Results

**Figure 3:**
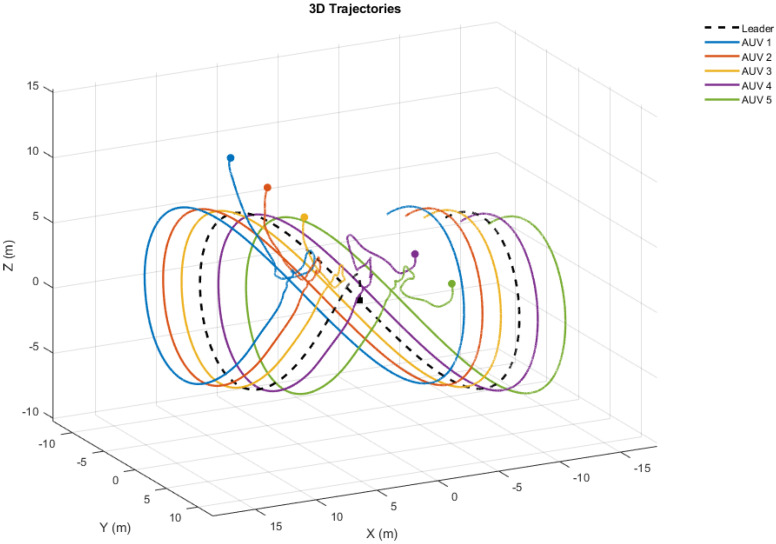
Trajectories of the BlueROV2 agents converging to the predefined positions around the leader’s trajectory.

As shown in Figure 3, the simulation begins with all five BlueROV2 vehicles initialized at

$$\begin{array}{cc} \eta_{1}\left(0\right) & ={\left[\;7,\;-7,\;10,\;\frac{\pi }{4},\;-\frac{\pi }{4}\;\right]}^{T},\\ \eta_{2}\left(0\right) & ={\left[\;5,\;-5,\;8,\;\frac{\pi }{5},\;-\frac{\pi }{5}\;\right]}^{T},\\ \eta_{3}\left(0\right) & ={\left[\;3,\;-3,\;6,\;\frac{\pi }{6},\;-\frac{\pi }{6}\;\right]}^{T},\\ \eta_{4}\left(0\right) & ={\left[\;-3,\;3,\;4,\;-\frac{\pi }{6},\;\frac{\pi }{6}\;\right]}^{T},\\ \eta_{5}\left(0\right) & ={\left[\;-5,\;5,\;2,\;-\frac{\pi }{5},\;\frac{\pi }{5}\;\right]}^{T}, \end{array}$$

where each state vector $\eta_{i}\left(0\right)={\left[\;x_{i}\left(0\right),\;y_{i}\left(0\right),\;z_{i}\left(0\right),\;\phi_{i}\left(0\right),\;\psi_{i}\left(0\right)\;\right]}^{T}$ corresponds to the initial position and orientation of AUV *i*. We can observe that all follower agents’ positions converge to the predefined positions around the leader’s trajectory, demonstrating formation tracking.

**Figure 4:**
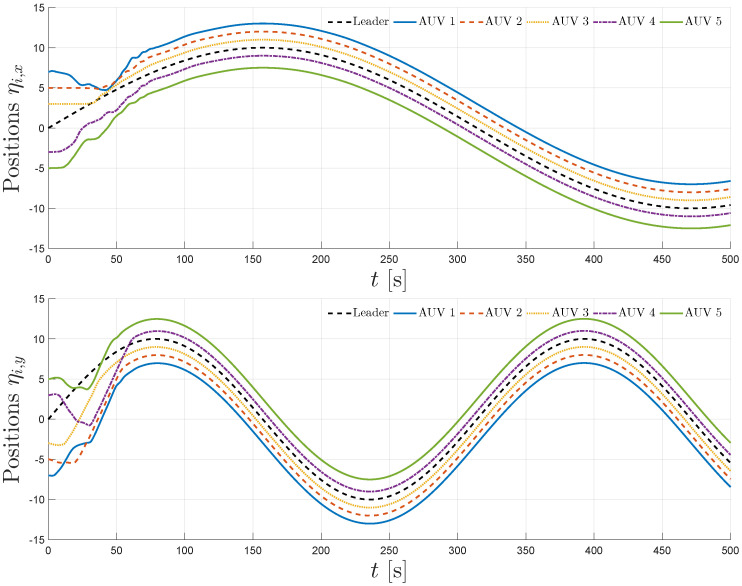

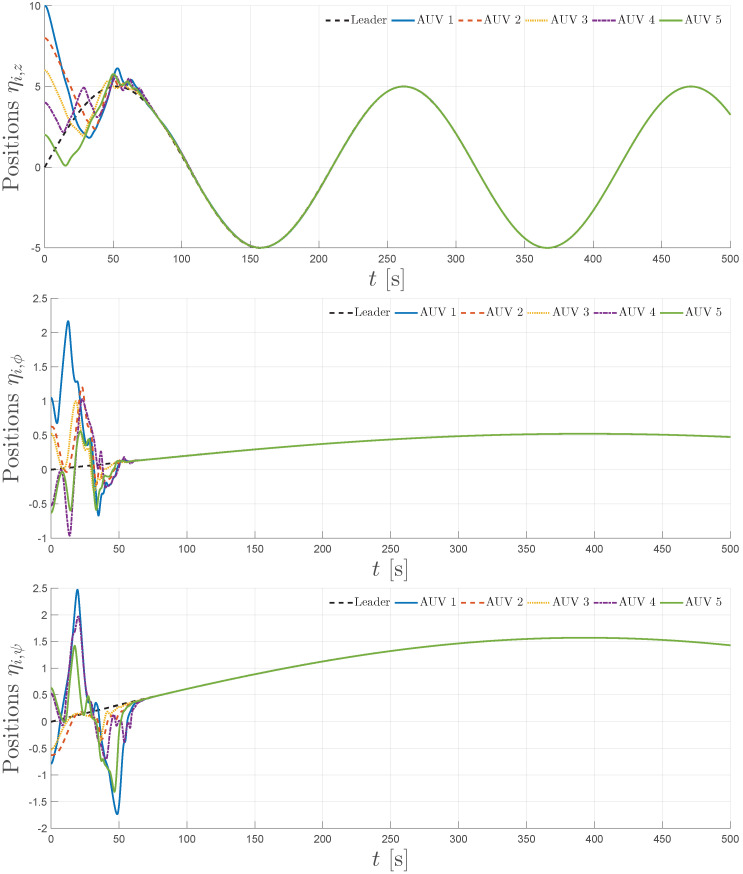
Leader and follower position trajectories for x,y,z,ϕ, and ψ.

**Figure 5:**
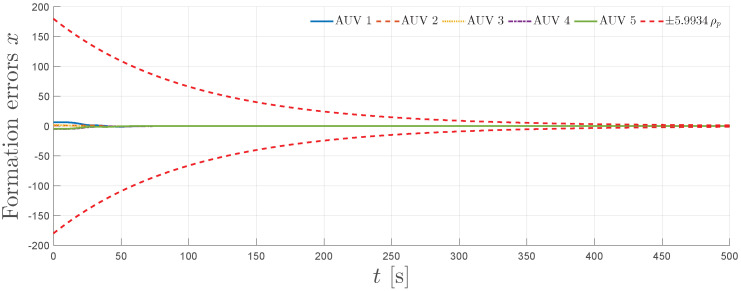

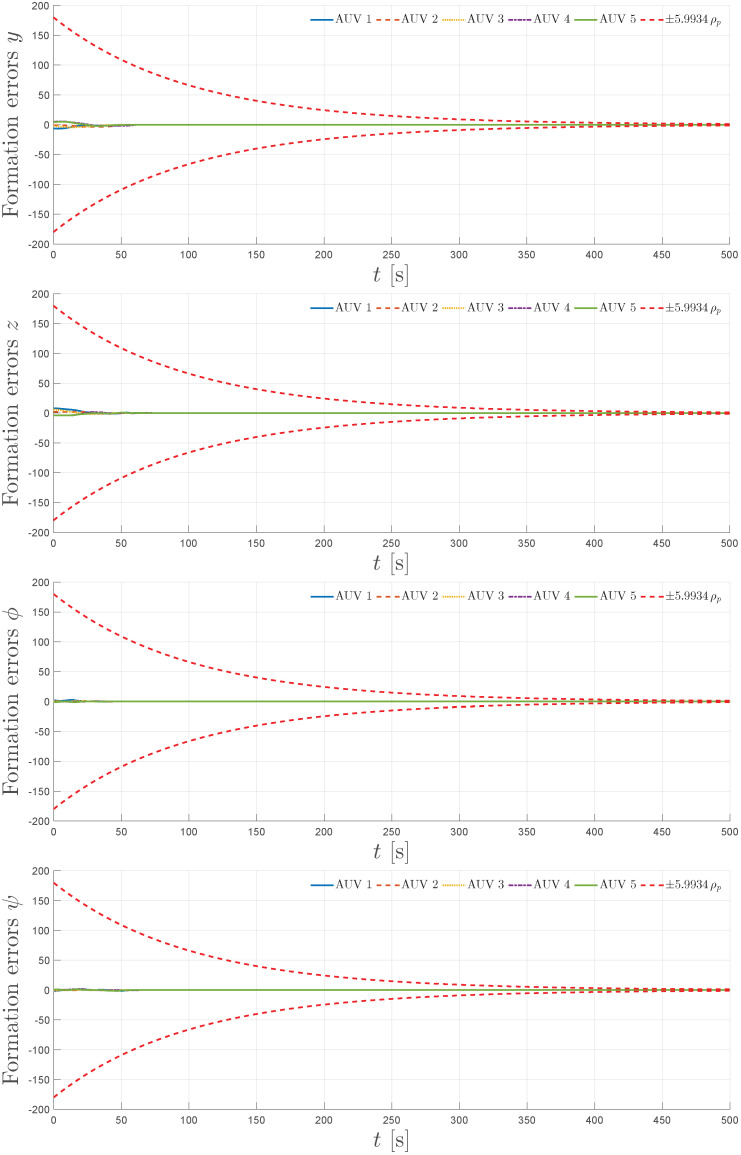
Formation errors for x,y,z,ϕ, and ψ and the scaled prescribed performance functions $\pm 5.9934\rho_{p}$.

**Figure 6:**
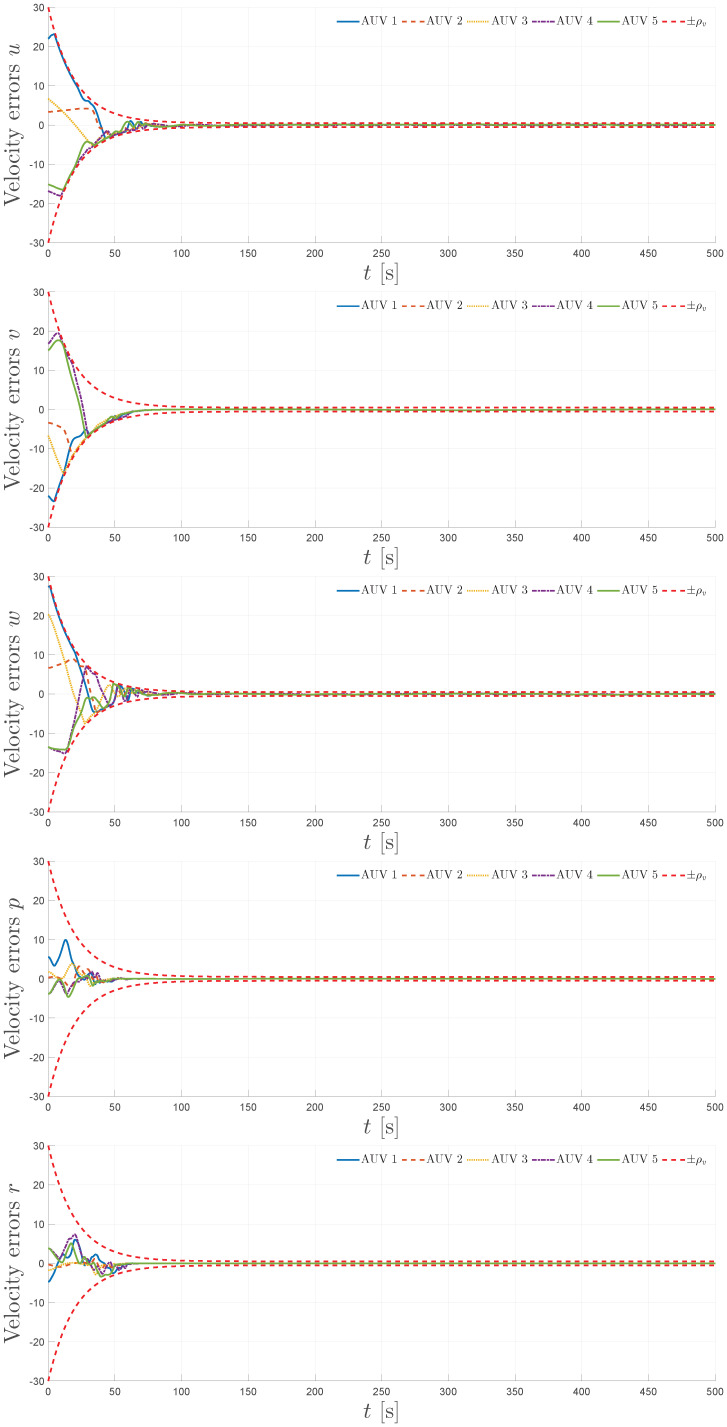
Velocity errors for u,v,w,p, and *r* and the prescribed performance functions.

**Figure 7:**
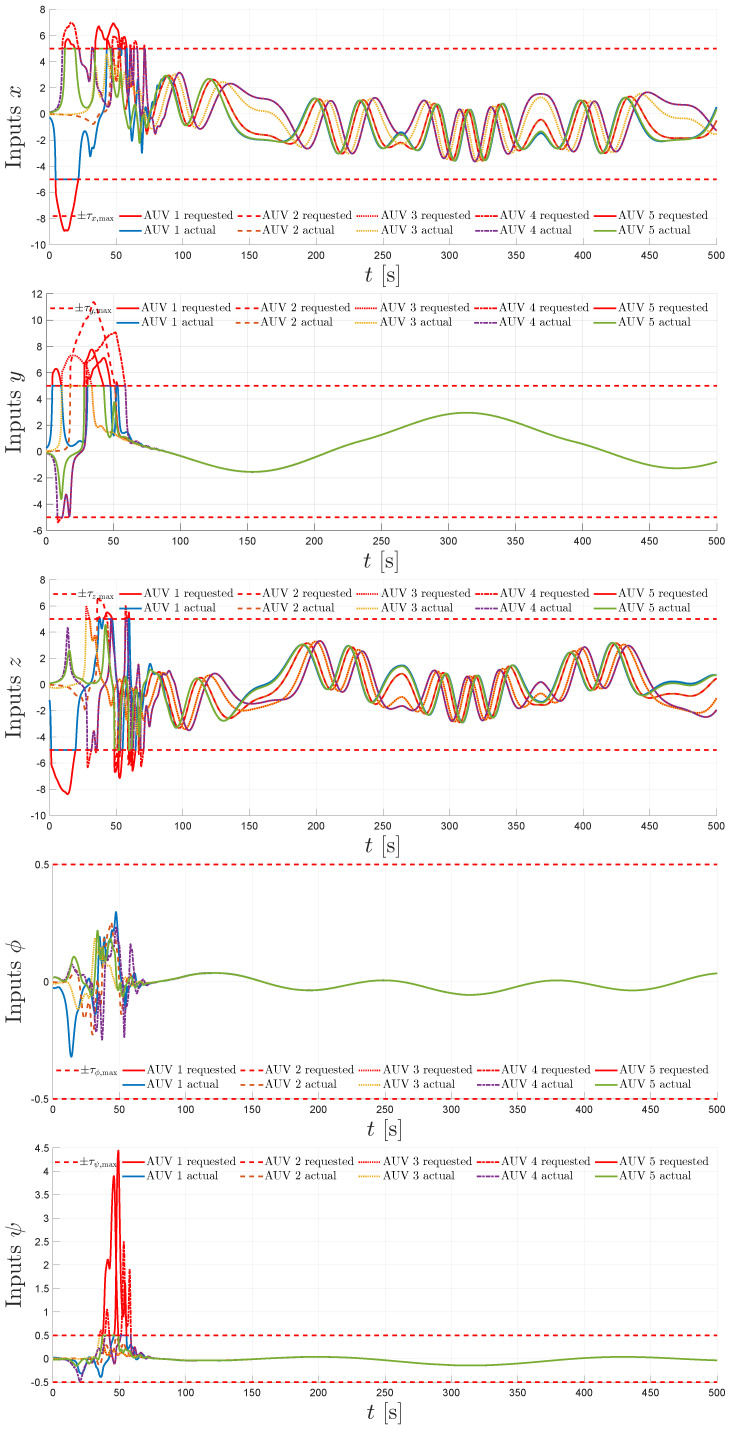
Control efforts: requested, actual, and their saturation levels for x,y,z,ϕ, and ψ.

**Figure 8:**
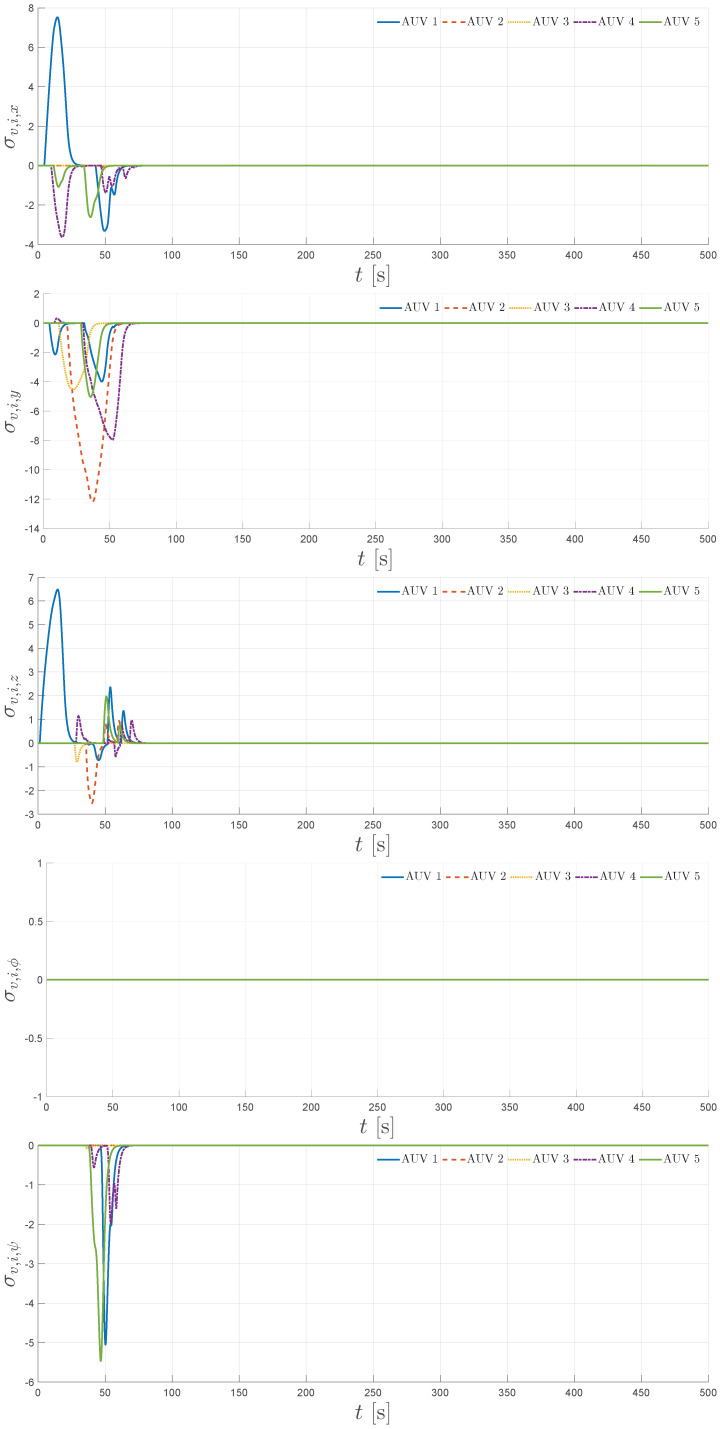
Virtual velocity reference modification signals for x,y,z,ϕ, and ψ.

The simulation results are summarized in Figure 4, Figure 5, Figure 6, Figure 7 and Figure 8. Figure 4 illustrates the trajectories of the leader and all five followers in the *x*, *y*, *z*, ϕ, and ψ coordinates. The leader follows the predefined sinusoidal trajectory, while the followers converge smoothly to their respective formation offsets, confirming successful prescribed performance formation tracking.

Figure 5 shows the evolution of the formation errors for all five followers in the *x*, *y*, *z*, ϕ, and ψ directions. The red dashed curves represent the scaled prescribed performance formation bounds, depicted as $\pm \kappa_{N}\rho_{p}$, with $\kappa_{N}:=\frac{\sqrt{N}}{\sigma_{\min}\left(L+B\right)}=5.9934$, as dictated by the results of Theorem 1. As observed, all formation errors remain strictly within the bounds for the entire simulation horizon, demonstrating the guaranteed transient and steady-state performance.

Figure 6 presents the velocity errors for the surge *u*, sway *v*, heave *w*, roll *p*, and yaw *r* components. Again, the red dashed curves correspond to the prescribed performance bounds for the modified velocities, depicted as $\pm \rho_{v}$. The errors remain within these envelopes, showing that the control law achieves stable and bounded modified velocity tracking.

The control inputs are shown in Figure 7. The requested efforts are contrasted against the actual applied efforts, which remain within the saturation limits. The ability of the proposed controller to handle hard input constraints without the loss of formation tracking is confirmed.

Finally, Figure 8 shows the virtual velocity modification signals $\sigma_{v,i,j}$ for all followers i=1,…,5 in the *x*, *y*, *z*, ϕ, and ψ dimensions. These signals are activated whenever requested inputs exceed the saturation limits, smoothly modifying the virtual velocity reference and thereby preserving stability and performance. Together, these results confirm that the proposed distributed control scheme achieves robust prescribed performance formation tracking under realistic six-DOF underwater dynamics, limited communication, and input saturation.

## 6. Conclusions

This paper presented a novel distributed prescribed performance control framework for the formation tracking of uncertain Euler–Lagrange multi-agent systems under input saturation. The approach introduces a distributed virtual velocity reference modification mechanism that dynamically adjusts each agent’s reference in the presence of actuator saturation, ensuring internal stability and preserving prescribed performance bounds. A rigorous analysis established the boundedness of all closed-loop signals and derived explicit feasibility conditions on control limits. The method is continuous, approximation-free, and requires only local neighbor and leader information, making it computationally efficient and suitable for real-time implementation in resource-constrained robotic systems.

High-fidelity six-DOF simulations with multiple BlueROV2 underwater vehicles demonstrated that the proposed controller achieves precise formation tracking while respecting input constraints, even with partial leader visibility and nonlinear hydrodynamic effects.

Future studies will focus on experimental validation in real underwater environments and an extension to time-varying communication topologies.

## Data Availability

Data are contained within the article.
